# Continued Circulation of Highly Pathogenic H5 Influenza Viruses in Vietnamese Live Bird Markets in 2018–2021

**DOI:** 10.3390/v15071596

**Published:** 2023-07-21

**Authors:** Lizheng Guan, Lavanya Babujee, Victoria L. Browning, Robert Presler, David Pattinson, Hang Le Khanh Nguyen, Vu Mai Phuong Hoang, Mai Quynh Le, Harm van Bakel, Gabriele Neumann, Yoshihiro Kawaoka

**Affiliations:** 1 Influenza Research Institute, Department of Pathobiological Sciences, School of Veterinary Medicine, University of Wisconsin-Madison, Madison, WI 53711, USA; lizheng.guan@wisc.edu (L.G.); lavanya.babujee@wisc.edu (L.B.); torey.browning@wisc.edu (V.L.B.); robert.presler@wisc.edu (R.P.); david.pattinson@wisc.edu (D.P.); 2 National Institute of Hygiene and Epidemiology, Hanoi 100000, Vietnam; nlkh@nihe.org.vn (H.L.K.N.); hvmp@nihe.org.vn (V.M.P.H.); ltqm@nihe.org.vn (M.Q.L.); 3 Department of Genetics and Genomic Services, Icahn School of Medicine at Mount Sinai, New York, NY 10029, USA; harm.vanbakel@mssm.edu; 4 Division of Virology, Department of Microbiology and Immunology, International Research Center for Infectious Diseases, The Institute of Medical Science, University of Tokyo, Tokyo 108-8639, Japan; 5 Research Center for Global Viral Diseases, National Center for Global Health and Medicine, Tokyo 162-8655, Japan; 6 The University of Tokyo Pandemic Preparedness, Infection and Advanced Research (UTOPIA) Center, Tokyo 108-8639, Japan

**Keywords:** influenza, surveillance, Vietnam

## Abstract

We isolated 77 highly pathogenic avian influenza viruses during routine surveillance in live poultry markets in northern provinces of Vietnam from 2018 to 2021. These viruses are of the H5N6 subtype and belong to HA clades 2.3.4.4g and 2.3.4.4h. Interestingly, we did not detect viruses of clade 2.3.4.4b, which in recent years have dominated in different parts of the world. The viruses isolated in this current study do not encode major determinants of mammalian adaptation (e.g., PB2-E627K or PB1-D701N) but possess amino acid substitutions that may affect viral receptor-binding, replication, or the responses to human antiviral factors. Several of the highly pathogenic H5N6 virus samples contained other influenza viruses, providing an opportunity for reassortment. Collectively, our study demonstrates that the highly pathogenic H5 viruses circulating in Vietnam in 2018–2021 were different from those in other parts of the world, and that the Vietnamese H5 viruses continue to evolve through mutations and reassortment.

## 1. Introduction

Highly pathogenic avian influenza viruses of the H5 subtype were first detected in Vietnam in 2001 and have caused multiple outbreaks in poultry in that country. Since their emergence in 1996, highly pathogenic avian influenza viruses have undergone substantial evolution through mutations in HA (hemagglutinin, the viral receptor-binding protein and major viral antigen), resulting in multiple clades and subclades. In addition, frequent reassortment with low pathogenicity avian influenza viruses has created multiple genotypes involving all the viral RNA segments except HA. Since 2014, highly pathogenic avian influenza viruses of clade 2.3.4.4 have been dominant in Southeast Asia, Europe, and Africa. In Vietnam, viruses of this clade were first detected in 2014 [1]. Further evolution of clade 2.3.4.4 viruses resulted in clade 2.3.4.4b H5N6 and H5N8 viruses, which have caused major outbreaks in poultry and wild birds in Europe since 2016. A novel clade 2.3.4.4b virus of the H5N1 subtype emerged in Europe in the fall of 2020 [2,3] and these viruses have caused outbreaks of an unprecedented scale in Europe, Asia, the Americas, and Africa [2,3,4]. Despite the global dominance of clade 2.3.4.4b viruses in recent years in most parts of the world, clade 2.3.4.4b viruses (of the H5N6 subtype) were detected in Vietnam in 2014, but not since then. Our surveillance activities in Vietnam in 2016–2017 identified highly pathogenic avian influenza viruses of clades 2.3.2.1c, 2.3.4.4f, and 2.3.4.4g in apparently healthy birds [5], and an analysis of sequences deposited in GISAID (Global Initiative on Sharing Avian Influenza Data www.gisaid.org) revealed highly pathogenic avian influenza viruses of clades 2.3.2.1c, 2.3.4.4, 2.3.4.4e, 2.3.4.4g, and 2.3.4.4h in Vietnam in 2016–2017. Here, we report the results of our surveillance activities in Vietnam from 2018 through to early 2021, which resulted in the isolation of 77 highly pathogenic H5N6 influenza viruses of clades 2.3.4.4g and 2.3.4.4h.

## 2. Materials and Methods

### 2.1. Virus Isolation and Identification

As part of its routine surveillance activities, the National Institute of Health and Epidemiology of Vietnam collected oropharyngeal and cloacal swab samples from apparently healthy birds in live bird markets in Hanoi and Qaung Ninh provinces, in northern Vietnam. From 2018 through to early 2021, 2430 samples were collected. The samples were placed in transport medium (DMEM containing 0.15% BSA, 100 IU/mL penicillin-streptomycin, 0.5 μg/mL amphotericin B, 100 μg/mL gentamicin, 20 μg/mL ciprofloxacin, and 0.02 M HEPES) and stored for further processing.

Up to 10 samples collected in the same location on the same day from the same species were pooled and inoculated into 9–11-day-old specific pathogen-free (SPF) eggs, and then incubated at 35 °C for 24–48 h. Following incubation, the allantoic fluid was tested for hemagglutination activity. Individual samples from pools in which hemagglutination was observed were each inoculated into two SPF eggs, and then tested for hemagglutination activity. All hemagglutination-positive samples were deep-sequenced.

### 2.2. Viral Genomic Sequence Analysis

RNA was extracted using the MagMAXTM-96 Viral RNA Isolation Kit (ThermoFisher). Extracted RNA was amplified by RT-PCR using the following oligonucleotides: F1 (5′-GTTACGCGCCAGCAAAAGCAGG); F2 (5′-GTTACGCGCCAGCGAAAGCAGG); and R1 (5′-GTTACGCGCCAGTAGAAACAAGG). The amplified double-stranded cDNAs were purified with 0.45× volume of AMPure XP beads (Beckman Coulter) and fragmented by acoustic shearing (0.5–1 μg) on a Bioruptor Pico sonicator (Diagenode) with an average fragment size of 150 bp. Library preparation for next-generation sequencing used the NEBNext DNA library prep modules for Illumina (New England Biolabs), and consisted of end repair, phosphorylation, A-tailing, and adaptor ligation, followed by additional PCR amplification.

The Illumina MiSeq demultiplexed reads were processed and assembled using the Iterative Refinement Meta-assembler (IRMA v. 1.0.2) [6]. Internal gene sequences were obtained from the primary assembly, whereas the HA and NA sequences were obtained from the secondary assembly. The default IRMA FLU parameters were used, with the following exceptions: LABEL was used for read-sorting, the residual assembly factor was set to 400 for the secondary assembly, and reference elongation was prevented. All sequence data were preprocessed, IRMA was run, and a downstream analysis was conducted by using version v0.9.9 of a snakemake workflow available at https://github.com/IRI-UW-Bioinformatics/flu-ngs/releases/tag/v0.9.9 (accessed on 8 August 2022). Database accession numbers are listed in Table 1.

### 2.3. Phylogenetic Analysis

Influenza virus sequences from avian hosts collected in Asia between 1 January 2015 and 17 January 2023 were obtained from GISAID (www.gisaid.org; accessed for HA on 17 January 2023; accessed for all other influenza viral genes between 2 June 2023 and 7 June 2023) (Supplemental Table S1). For HA, sequences from all H5 viruses were downloaded. For NA, all N6 sequences were downloaded. For the internal genes PB2, PB1, and NS1, all influenza A virus sequences were used. An R script was used to randomly select 10% of HA sequences (320), or about 250 sequences (225–275) for each of the other segments for inclusion in phylogenetic trees. After random selection, duplicate and non-full-length sequences were removed. For NS1, only allele A sequences were included. The sequences were aligned using MAFFT [7] through the Geneious Prime software package. Phylogenetic trees were constructed using RAxML-ng [8] with the GTR+I+G4 substitution model with up to 1000 bootstrap replicates, using MRE-based bootstopping [9]. Trees were visualized using ggtree [10]. A/goose/Guangdong/1/1996 was used as an outgroup for rooting the HA tree; the other trees are unrooted.

### 2.4. Biosafety Statement

The isolation of the surveillance samples was conducted in an enhanced biosafety level 3 (BSL 3+) laboratory at the University of Wisconsin–Madison. The RNA extraction/inactivation protocol was approved by the University of Wisconsin–Madison’s Institutional Biosafety Committee (IBC) after conducting a risk assessment in the Office of Biosafety. All experiments were approved by the University of Wisconsin–Madison’s IBC. This manuscript was reviewed by the University of Wisconsin–Madison Dual Use Research of Concern (DURC) Subcommittee. This review was conducted in accordance with the United States Government September 2014 DURC Policy. The DURC Subcommittee concluded that the studies described herein do not meet the criteria of Dual Use Research of Concern (DURC).

## 3. Results

### 3.1. Isolation and Identification of Avian Influenza Viruses from Vietnamese Live Bird Markets in 2018 through Early 2021

**Figure 1:**
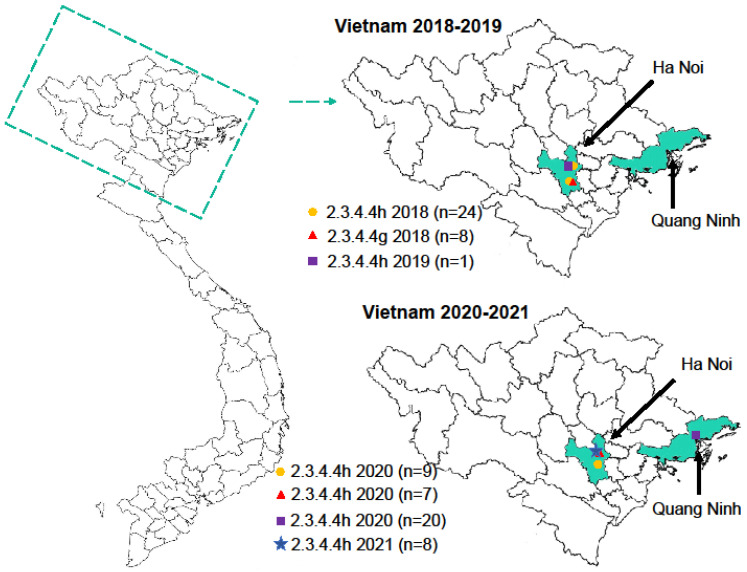
Geographic locations of surveillance activities in Vietnamese live bird markets from 2018 to 2021. Left, map of Vietnam with province borders. Right, magnification of the northern part of Vietnam. The provinces where the samples were isolated are shown in aquamarine. The virus clades are indicated by different colors and symbols with the number of isolates in parenthesis. The symbols in the map indicate the different locations at which viruses were collected.

We have been conducting routine surveillance in Vietnamese live bird markets since 2009, resulting in the isolation of multiple highly pathogenic H5 influenza viruses [5,11]. Here, we collected 590 (2018), 840 (2019), 790 (2020), and 210 (early 2021) oropharyngeal and cloacal swab samples from apparently healthy chickens and ducks at live bird markets in the northern provinces of Vietnam (Figure 1). Pooled samples (comprising up to 10 samples collected on the same day, in the same location, and from the same species) were inoculated into SPF-embryonated chicken eggs and tested for hemagglutination activity. If a batch was hemagglutination-positive, all samples from this batch were individually inoculated into SPF-embryonated chicken eggs and tested for their hemagglutination activity. All hemagglutination-positive samples were deep-sequenced to assess their subtype and genome composition. In total, 77 highly pathogenic avian influenza viruses of the H5N6 subtype were detected, some as a mixture with other influenza viruses (Table 1). In addition, we isolated 334 low pathogenic avian influenza viruses of various subtypes, primarily H6N6 (193 isolates), H3N2 (50 isolates), H9N2 (23 isolates), H4N2 (17 isolates), H4N6 (16 isolates), and H6N2 (13 isolates); these viruses were not analyzed further in the current study.

### 3.2. Phylogenetic Analysis of Highly Pathogenic H5N6 Viruses

We phylogenetically analyzed the HA and NA genes, which encode the viral surface glycoproteins and major or minor viral antigens, respectively, as well as the polymerase (PB2, PB1, PA) and interferon antagonist (NS1) viral genes whose products affect influenza virus pathogenicity. All segments were analyzed individually.

**Figure 2:**
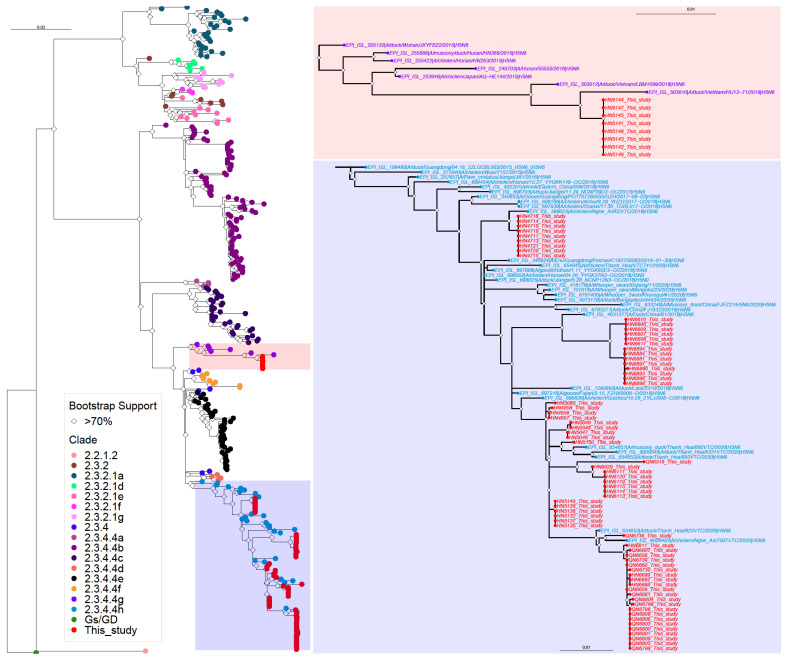
Phylogenetic analysis of the HA genes of highly pathogenic H5N6 influenza viruses isolated in Vietnam from 2018 to 2021. Left, schematic phylogenetic tree without virus names (for the phylogenetic tree with virus names, see Supplemental Figure S1). The sections of the phylogenetic tree containing the highly pathogenic H5N6 viruses from this study are shown with higher magnification and virus names on the right. Viruses isolated in this study are shown in red. All other viruses are color-coded based on the HA clade. Bootstrap values greater than 70% are indicated by open diamonds.

We determined the phylogeny of 300 randomly selected H5 HA viral sequences downloaded from GISAID that were collected in Asia between 1 January 2015 and 17 January 2023, plus A/goose/Guangdong/1/1993 for the outgroup. The HA genes of the isolated H5N6 viruses were found within clade 2.3.4.4g and 2.3.4.4h viruses (Table 1, Figure 2 and Figure S1). The HA genes of the clade 2.3.4.4g viruses isolated in our study were identical at the amino acid level and most closely related to the HA genes of H5N6 viruses collected in Vietnam, China, and Japan in 2015–2019 (Figure 2 and Figure S1). The HA genes of clade 2.3.4.4h viruses fell into several sub-groups, all of which were closely related to those of the Vietnamese and Chinese H5 viruses isolated in 2015–2020 (Figure 2 and Figure S1).

**Figure 3:**
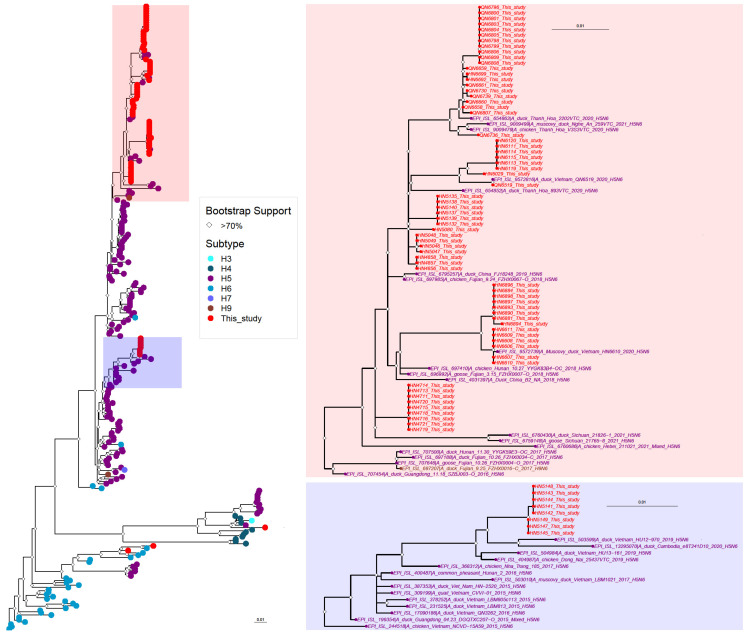
Phylogenetic analysis of the NA genes of highly pathogenic H5N6 influenza viruses isolated in Vietnam from 2018 to 2021. Left, schematic phylogenetic tree without virus names (for the phylogenetic tree with virus names, see Supplemental Figure S2). The branches of the phylogenetic tree containing the highly pathogenic H5N6 viruses from this study are shown with higher magnification and virus names on the right. Viruses isolated in this study are shown in red. All other viruses are color-coded based on the HA subtype. Bootstrap values greater than 70% are indicated by open diamonds.

All highly pathogenic H5 viruses isolated in our study possessed NA genes of the N6 subtype (Table 1). To assess their phylogenetic relationships, we downloaded all N6 NA sequences collected in Asia between 1 January 2015 and 17 January 2023 and randomly selected about 250 NA sequences to generate a phylogenetic tree. The NA genes of the clade 2.3.4.4g and 2.3.4.4h viruses were in different branches of the phylogenetic tree (Figure 3 and Figure S2), indicating co-segregation with the HA genes. The NA genes of most clade 2.3.4.4h viruses were closely related to each other, but not identical (Figure 3 and Figure S2). Three NA genes of clade 2.3.4.4h viruses clustered most closely with the NA genes of avian H4 or H6 viruses (Supplemental Figure S3); however, the respective isolates contained a second influenza virus.

Influenza viruses undergo frequent reassortment with low pathogenic avian influenza viruses, resulting in multiple genotypes. To assess the phylogenetic origins of the H5N6 virus polymerase and NS1 genes, we downloaded the respective sequences of all Asian influenza A viruses collected between 1 January 2015 and 17 January 2023 (for NS1, only sequences of allele A were included in the analysis). Again, about 250 randomly selected sequences were used to generate phylogenetic trees (Supplemental Figures S3–S6). The PB2 genes fell into two larger subgroups that did not co-segregate with the HA clade of the respective virus (Supplemental Figure S3). Most of the PB2 genes were closely related to those of other highly pathogenic H5 influenza viruses; however, one group of H5N6 viruses isolated here possessed PB2 genes that were closely related to the PB2 gene of a Chinese H7N4 virus isolated from migratory wild bird in 2019, indicating reassortment with a low pathogenic avian influenza virus. The PB1 genes of the viruses characterized here formed several groups in the phylogenetic tree (Supplemental Figure S4). Based on our results that one group comprised clade 2.3.4.4g viruses, we conclude that the HA, NA, and PB1 genes of these viruses co-segregated. A second group was formed by viruses isolated in late December 2020 whose PB2 genes were closely related to that of a low pathogenic avian influenza virus (see Supplemental Figure S3). The PB1 genes of these viruses resembled the PB1 genes of low pathogenic avian influenza virus, further indicating recent reassortment. For the remaining viruses, most of the PB1 genes were relatively similar to each other and related to other highly pathogenic H5 influenza viruses. The PA genes of the viruses characterized in our study formed several different groups in the phylogenetic tree that did not correlate with the HA clade of the respective virus (Supplemental Figure S5). Two of these groups were closely related to low pathogenic avian influenza viruses of different subtypes, again suggesting recent reassortment. The NS1 genes of our Vietnamese H5N6 viruses were segregated based on the HA clade (Supplemental Figure S6), indicating that the HA, NA, PB1, and NS1 genes of the 2.3.4.4g viruses co-segregated.

### 3.3. Comparison of the Amino Acid Consensus Sequences of Highly Pathogenic H5N6 Viruses

To characterize the isolated H5N6 viruses in more detail, we first assembled and compared their consensus amino acid sequences. The viral HA protein is a major determinant of influenza virus pathogenicity. All highly pathogenic avian influenza viruses analyzed here encoded multiple basic amino acids (i.e., ‘RERRRKR’) at the HA cleavage site (Supplemental Table S2); this sequence motif is recognized by ubiquitous proteases and enables systemic virus spread, resulting in high pathogenicity. At amino acid positions 222/224 (based on the amino acid numbering of mature H5 HA protein), the highly pathogenic H5 viruses isolated in our study encoded HA-222Q/224G (Supplemental Table S2), which confer binding to α2,3-linked sialic acids [12], that is, avian-type receptors. Several other amino acid substitutions may increase binding to α2,6-linked sialic acids (i.e., human-type receptors), including HA-S120N [13], -S133A [14], -△L129/I151T [15], -T156A [13,16,17,18], -N182K [19,20], -K189R/T [13,21], -Q192R [19,20,22], -Q192H [15], -S223N [17] or -P235S [15]. In addition, the amino acid at HA position 223 also affects influenza viral antigenicity [13,23]. The viruses isolated here encoded substitutions at several of these positions, specifically HA-S133A, -T156A, and in some cases, -P235R (Supplemental Table S2); however, the consequences of these amino acid changes for the Vietnamese H5N6 viruses are unknown.

The viral neuraminidase (NA) gene encodes a sialidase that releases infecting viruses from sialic acids during the infection process and facilitates the release of newly generated virus protein from infected cells late in infection. All viruses isolated in this study possessed NAs of the N6 subtypes, whereas our previous surveillance activities resulted in the isolation of H5 viruses of the N1 and N6 subtypes [5]. Most of the analyzed N6 NA proteins possessed a deletion of 11 amino acids in the NA stalk (amino acid positions 58–68 of NA) (Supplemental Table S2), which has been detected in H5N6 viruses since 2015 [24] and increases virus replication in cultured cells [25]. However, three samples (A/duck/Vietnam/HN5150/2018, A/Muscovy duck/Vietnam/HN6698/2020, A/Muscovy duck/Vietnam/HN6811/2020), all of which contained a second influenza virus, possessed full-length NA proteins. NA is the target of neuraminidase inhibitors (i.e., the most commonly prescribed antivirals for influenza viruses) and the NA-E119D [26], -E119V/I222L [27], -I222V [28], -A246V [26], -R292K [26], and -R371K [26] mutations reduce the efficacy of these inhibitors. The H5N6 viruses assessed here did not encode these mutations (note that positions 119, 222, 246, 292, and 371 refer to positions 119, 223, 247, 293, and 372, respectively, in our alignment; Supplemental Table S2).

The pathogenicity and host range of influenza viruses is also determined by the PB2 protein, a component of the viral polymerase complex. The most prominent mammalian-adapting amino acid changes are PB2-E627K [29,30] and -D701N [31]; none of the viruses characterized here encoded either of these mammalian-adapting markers (Supplemental Table S2). Other substitutions in PB2 that increase pathogenicity or replication include PB2-I147T/K339T/A588T [32], -E158G [33], -T271A [34], -Q590S [35], -Q591K/R [35], and -S714R [36], but none of these substitutions was encoded by the viruses isolated in this study. We did, however, detect the PB2-K123E substitution in most of the viruses isolated in our study; this substitution has been shown to increase the pathogenicity of an H7N7 virus in chickens [37]. A lysine residue at PB2-251 (detected in some of the viruses characterized here) was reported to increase the replication and pathogenicity of an avian-like H1N1 swine influenza virus in mice [38]. Moreover, the viruses in our study encoded different amino acids at position 292; these substitutions may affect virus replication and transmission [39,40,41]. Several studies have shown that the amino acid at PB2-526 affects viral polymerase activity [42,43,44]; most viruses in our study encoded PB2-526K (associated with lower polymerase activity), although some encoded PB2-526R (associated with increased polymerase activity) (Supplemental Table S2). In addition, PB2-588V (encoded by most viruses analyzed here; Supplemental Table S2) increases viral polymerase activity and replication compared with viruses encoding PB2-588A [45]. Finally, several of the Vietnamese isolates described here encoded PB2-660R or -702R., which increase the viral polymerase activity compared with PB2-660K or -702K, respectively [43,46].

The PB1 gene encodes another component of the viral polymerase complex. Consequently, several mutations in this protein can affect the viral polymerase activity, including PB1-E180D [47], -T296R [48], -M317V [47], -N375S [49], -V473L [50], -K480R [51], -S524G [52], -K577E [53], and -P598L [50]. All viruses isolated in our study encoded PB1-598L and some encoded -317V or -375S (Supplemental Table S2); at the other amino acid positions listed above, the Vietnamese H5N6 viruses encoded amino acids associated with lower viral polymerase activity.

The PA protein is also part of the influenza virus polymerase complex and several amino acid substitutions in this protein affect the viral polymerase activity and/or virulence, including PA-E18G/S388R/A448E [54], -T85I [55], -D101G [56], -G186S [55], -P224S [57], -K237E [56], -L336M [55], -A343S alone or -A343S/D347E [58], -K356R [59], and -K391R [60]. At most of these amino acid positions, the viruses characterized here encoded an amino acid associated with lower polymerase activity and/or virulence; however, the viruses analyzed in our study encoded PA-224S and -237E, and some also encoded -K356R and -K391R (Supplemental Table S2). At several amino acid positions, namely, PA-100, -241, -356, and -404, the Vietnamese H5N6 viruses described here possessed amino acids that are characteristic of human influenza viruses, based on computational analyses [61,62,63,64]. However, without experimental testing, we do not know the functional consequences of these amino acid substitutions.

The NS viral RNA segment encodes, in a contiguous reading frame, the NS1 protein, whose primary function is to counteract the innate immune response of the host [65]. Most recent highly pathogenic avian H5 viruses possess a deletion of five amino acids (from positions 80–84) in their NS1 protein, which was also detected in most of the viruses sequenced here (Supplemental Table S2). This deletion increases the pathogenicity of H5N1 viruses in mice compared with viruses encoding a full-length NS1 protein [66]. All viruses that did not encode the NS1∆80-84 deletion were found together with one or two other influenza viruses (Supplemental Table S2), suggesting that the consensus NS1 sequence reflects that of the low pathogenic avian influenza virus in the sample. Another sequence motif that contributes to high H5 virus pathogenicity in mice is a PDZ domain-binding motif with the sequence “ESEV” at the C-terminus of NS1 [67], which was detected in all viruses sequenced here, except for samples that contained two influenza viruses (Supplemental Table S2).

The PB1-F2 protein (encoded by an alternate open reading frame in the PB1 viral RNA segment) modulates the severity of bacterial infections, type I IFN responses, inflammasome function, and the induction of apoptosis (reviewed in [68,69]). All clade 2.3.4.4g viruses sequenced here encoded a full-length PB1-F2 protein of 90 amino acids (Supplemental Table S2). The clade 2.3.4.4h virus isolates fell into two groups encoding a full-length PB1-F2 protein (with sequences substantially different from those of clade 2.3.4.4g viruses) or truncated versions of 11 or 34 amino acids in length (which have been reported for other highly pathogenic H5 viruses) (Supplemental Table S2). Four sequences (all derived from samples that contained two influenza viruses) encoded the PB1-F2-N66S substitution, which increases influenza virulence [70] (Supplemental Table S2).

### 3.4. Viral Subpopulations of Highly Pathogenic H5N6 Viruses

After establishing the consensus sequences of the H5N6 viruses isolated in Vietnam in 2018–2021, we used these sequences as references to assess viral subpopulations. The sequences of isolates containing multiple HA and/or NA subtypes, indicating the presence of more than one influenza A virus, were removed from the dataset for these analyses. In addition, upon examination of the PB2, PB1, PA, and NS1 genes, A/duck/Vietnam/HN5144/2018 and A/duck/Vietnam/HN5148/2018 were found to contain more than one influenza A virus. Therefore, these samples were excluded from all further analyses. For the subpopulation analysis, we considered only subpopulations detected in ≥3% of the sequence reads.

Four of the viruses characterized here encoded minor subpopulations of 11–33% at amino acid position 120 of HA (HA-120R/G) (Supplemental Table S3). Wang et al. [13] demonstrated that the HA-S120N mutation increases binding to α2,6-linked sialic acid. However, the receptor-binding specificity of the variants detected here is currently not known. Polymorphisms were also detected at several HA positions located at the rim of the receptor-binding site, including HA-128S/P, HA-134A/S, HA-136P/L, and HA-192K/N. The HA substitutions -S128L [71] and -P140L [72] affect HA stability, whereas -A134V and -Q192R (together with other substitutions) affect the receptor-binding specificity of H5N1 virus [19,20,22,73]. For one isolate (A/duck/Vietnam/QN6659/2020), an HA-223R/G polymorphism was detected at low frequency. This amino acid position is part of the receptor-binding site and substitutions at this position (although not the polymorphism detected here) may affect receptor-binding and antigenic properties [23]. Several of the Vietnamese H5N6 viruses possessed an HA-176W/L polymorphism; the tryptophan residue at this amino acid position is conserved among all HA subtypes.

No polymorphisms were detected at NA amino acid positions that affect sensitivity to NA inhibitors (Supplemental Table S3). At amino acid positions 407–410 (amino acid positions 418–421 for NA proteins without the deletion at positions 58–68), multiple isolates possessed subpopulations at low frequencies of 3–7%.

At amino acid position 253 of PB2, two isolates encoded both aspartic acid and minor subpopulations of asparagine (PB2-253D/N) (Supplemental Table S3). Several studies have shown that PB2-D253 increases influenza virus replication and pathogenicity [43,74,75]. Subpopulations were also detected for two isolates at amino acid 292 (PB2-292I/V), which may affect virus replication [39,40,41]. Moreover, several other sequence nucleotide polymorphisms were detected at high frequency (e.g., PB2-106I/T, -112P/S, and -153D/N).

Inspection of PB1 subpopulations revealed a PB1-105N/D polymorphism (Supplemental Table S3), which might affect virus replication because a substitution at this position (PB1-N109S) has been shown to increase influenza virus replicative ability [46]. Thirteen different isolates possessed a PB1-453A/V polymorphisms, but the biological consequences of these subpopulations are unknown. Moreover, 11 different isolates encoded a PB1-591V/D polymorphism. Interestingly, in a previous study, the PB1-V591I substitution conferred a temperature-sensitive phenotype to the live attenuated A/Leninggrad/134/17/57 (H2N2) virus [76].

The PA-85T/I polymorphic residue detected in one of the isolates (A/Muscovy duck/Vietnam/HN5139/2018; Supplemental Table S3) may affect virus replication because the PA-T85I substitution has been shown to increase the polymerase activity of an H7N9 virus [60] and PA-85I was shown to confer high replicative ability to a human pandemic influenza virus [55]. Virus replication may also be enhanced by the PA-127V/A polymorphism based on data showing that this substitution enhanced H5N1 virus growth in human cells [77]. The PA-K328E substitution increased virus replication in mini-replicons [78]; a PA-328K/E polymorphism was detected in one of the isolates characterized here (A/duck/Vietnam/QN6798/2020) and may thus affect the replicative ability of the virus. In addition, we detected the PA-669V/I or -699I/V polymorphisms in four isolates at high frequency.

In NP, relatively few polymorphisms were detected (Supplemental Table S3), none of which is known to affect virulence. The viruses characterized here also possessed a few sequence nucleotide polymorphisms in the M1 gene (Supplemental Table S3). Two of these may be associated with biological functions: the M1-T37A substitution increased the infectivity of an H9N2 influenza virus [79] by increasing M1 stability [80], and the M1-A227S substitution affected nucleocytoplasmic transport [81]. We currently do not know whether the M1-37A/T and M1-227A/T polymorphisms detected in this study similarly influence the respective H5N6 viruses. Relatively few polymorphisms (which are not known to affect biological functions) were also detected for M2 (Supplemental Table S3).

The NS1-E167(172)K (the number in parenthesis indicates the amino acid position of NS1 proteins not possessing a deletion at amino acid positions 80–84) substitution increases the stimulation of interferon-stimulated genes [82] and may therefore modulate NS1 interference with cellular immune responses. An NS1-167(172)E/K polymorphism was detected in one of the Vietnamese H5N6 isolates (A/duck/Vietnam/QN6805/2020), but at the relatively low frequency of 5% (Supplemental Table S3). The NS2 genes of the Vietnamese H5N6 viruses in our study possessed few polymorphisms (Supplemental Table S3). The NS2-E75G substitution has been reported to compensate for the mammalian-adapting function of PB2-627K [83], but we do not know whether the NS1-75E/D polymorphism detected in one of the Vietnamese H5N6 isolates (A/duck/Vietnam/QN6739/2020) has a similar effect.

In 2018–2021, we conducted routine surveillance in apparently healthy chickens and ducks at live bird markets in northern Vietnam, and isolated 77 highly pathogenic H5N6 influenza viruses of clades 2.3.4.4g and 2.3.4.4h. During this time, Vietnam was the only country in which clade 2.3.4.4g viruses were being detected, although viruses of clade 2.3.4.4h were being detected in China, Bangladesh, Laos, and Japan (https://www.offlu.org/wp-content/uploads/2021/01/OFFLU_summary_report_26_02_2020_Avian_Section.pdf; accessed on 12 June 2023; www.GISAID.org). Since 2016, clade 2.3.4.4b viruses have been dominant in several parts of the world. From 2016–2020, clade 2.3.4.4b viruses of the H5N6 and H5N8 subtypes dominated in Asia and Europe, and in late 2020 an H5N1 clade 2.3.4.4b virus emerged in Europe and spread rapidly throughout the continent. This virus has also caused multiple outbreaks in the Americas. Interestingly, we did not detect clade 2.3.4.4b viruses in Vietnam between 2018 and 2021. Our surveillance findings are consistent with data from GISAID, which has reported no clade 2.3.4.4b viruses in Vietnam since 2016 (except for three isolates in 2021). Collectively, these findings indicate that the highly pathogenic H5 viruses circulating in live poultry markets in Vietnam in 2018–2021 were different from those detected in other parts of the world.

Viruses of clade 2.3.2.1c were detected by us in Vietnam in 2016–2017 (5), but not during the current surveillance period. Our analysis of viruses deposited in GISAID revealed 28 viruses of clade 2.3.2.1c in Vietnam in 2018–2019, but none since then. However, the OFFLU Avian Influenza Report for the period of February–September 2020 (https://www.offlu.org/wp-content/uploads/2021/01/OFFLU-avian-25092020_removed.pdf; accessed on 12 June 2023) lists clade 2.3.2.1c viruses in Vietnam, suggesting that these viruses may continue to circulate at low levels in Vietnam.

The H5N6 viruses characterized here do not possess major markers of adaptation to humans, such as PB2-E627K or PB2-D701N. The HA proteins of some of the viruses characterized here encode amino acid changes that may affect receptor-binding specificity. Overall, however, recent highly pathogenic H5 viruses have maintained their preference for α2,3-linked sialic acids (i.e., avian-type receptors) [84,85]. The H5N6 viruses isolated in our study also encode several amino acids that may affect viral replicative ability and are not common among highly pathogenic H5 viruses isolated in Asia from 2015 to 2021, such as PB1-317V and PB1-375S. However, for these and several other amino acid substitutions detected in our analysis, we currently do not know whether they affect the virulence of the viruses isolated in our study.

Several of the samples isolated in our study contained a highly pathogenic H5N6 isolate together with one or two other influenza viruses. Given the substantial number of low pathogenic avian influenza viruses isolated during our surveillance activities, this presents multiple opportunities for reassortment and the generation of novel genotypes, which may result in altered properties. Ongoing surveillance of avian influenza viruses in Vietnam and other parts of the world is therefore important to monitor the evolution of these viruses.

## Figures and Tables

**Table 1:** Highly pathogenic H5 influenza viruses isolated in Northern Vietnamese live bird markets in 2018–early 2021.

Isolate	Subtype(s) *	Subclade	Location	Province	Collection Date	Accession Number
A/Muscovy duck/ Vietnam/HN4711/2018	H5N6	2.3.4.4h	Thuong Tin	Ha Noi	9 June 2018	EPI_ISL_17768813
A/Muscovy duck/ Vietnam/HN4713/2018	H5N6	2.3.4.4h	Thuong Tin	Ha Noi	9 June 2018	EPI_ISL_17768814
A/Muscovy duck/ Vietnam/HN4714/2018	H5N6	2.3.4.4h	Thuong Tin	Ha Noi	9 June 2018	EPI_ISL_17768815
A/Muscovy duck/ Vietnam/HN4715/2018	H5N6	2.3.4.4h	Thuong Tin	Ha Noi	9 June 2018	EPI_ISL_17768816
A/Muscovy duck/ Vietnam/HN4716/2018	H5N6	2.3.4.4h	Thuong Tin	Ha Noi	9 June 2018	EPI_ISL_17768817
A/Muscovy duck/ Vietnam/HN4718/2018	H5N6	2.3.4.4h	Thuong Tin	Ha Noi	9 June 2018	EPI_ISL_17768818
A/Muscovy duck/ Vietnam/HN4719/2018	H5N6	2.3.4.4h	Thuong Tin	Ha Noi	9 June 2018	EPI_ISL_17768819
A/Muscovy duck/ Vietnam/HN4720/2018	H5N6	2.3.4.4h	Thuong Tin	Ha Noi	9 June 2018	EPI_ISL_17768820
A/duck/Vietnam/HN4721/2018	H5N6	2.3.4.4h	Thuong Tin	Ha Noi	9 June 2018	EPI_ISL_17768803
A/Muscovy duck/ Vietnam/HN4856/2018	H5N6	2.3.4.4h	Thuong Tin	Ha Noi	10 August 2018	EPI_ISL_17768821
A/Muscovy duck/ Vietnam/HN4857/2018	H5N6	2.3.4.4h	Thuong Tin	Ha Noi	10 August 2018	EPI_ISL_17768822
A/Muscovy duck/ Vietnam/HN4858/2018	H5N6	2.3.4.4h	Thuong Tin	Ha Noi	10 August 2018	EPI_ISL_17768823
A/Muscovy duck/ Vietnam/HN5046/2018	H5N6	2.3.4.4h	Gia Lam	Ha Noi	29 October 2018	EPI_ISL_17768824
A/Muscovy duck/ Vietnam/HN5047/2018	H5N6	2.3.4.4h	Gia Lam	Ha Noi	29 October 2018	EPI_ISL_17768825
A/Muscovy duck/ Vietnam/HN5048/2018	H5N6	2.3.4.4h	Gia Lam	Ha Noi	29 October 2018	EPI_ISL_17768826
A/duck/Vietnam/HN5080/2018	H5N6	2.3.4.4h	Thuong Tin	Ha Noi	5 November 2018	EPI_ISL_17768806
A/Muscovy duck/ Vietnam/HN5132/2018	H5N6	2.3.4.4h	Thuong Tin	Ha Noi	5 December 2018	EPI_ISL_17768828
A/Muscovy duck/ Vietnam/HN5135/2018	H5N6	2.3.4.4h	Thuong Tin	Ha Noi	5 December 2018	EPI_ISL_17768829
A/Muscovy duck/ Vietnam/HN5137/2018	H5N6	2.3.4.4h	Thuong Tin	Ha Noi	5 December 2018	EPI_ISL_17768830
A/Muscovy duck/ Vietnam/HN5138/2018	H5N6	2.3.4.4h	Thuong Tin	Ha Noi	5 December 2018	EPI_ISL_17768831
A/Muscovy duck/ Vietnam/HN5139/2018	H5N6	2.3.4.4h	Thuong Tin	Ha Noi	5 December 2018	EPI_ISL_17768832
A/Muscovy duck/ Vietnam/HN5140/2018	H5H9N2N6	2.3.4.4h	Thuong Tin	Ha Noi	5 December 2018	N/A
A/Muscovy duck/ Vietnam/HN5049/2018	H5N6	2.3.4.4h	Gia Lam	Ha Noi	5 December 2018	EPI_ISL_17768827
A/duck/Vietnam/HN5150/2018	H5H6N6	2.3.4.4h	Thuong Tin	Ha Noi	5 December 2018	N/A
A/Muscovy duck/ Vietnam/HN6029/2019	H5H6N6N8	2.3.4.4h	Gia Lam	Ha Noi	30 December 2019	N/A
A/Muscovy duck/ Vietnam/HN6111/2020	H5N6	2.3.4.4h	Thuong Tin	Ha Noi	10 February 2020	EPI_ISL_9572812
A/Muscovy duck/ Vietnam/HN6113/2020	H5N6	2.3.4.4h	Thuong Tin	Ha Noi	10 February 2020	EPI_ISL_9572787
A/Muscovy duck/ Vietnam/HN6114/2020	H5N6	2.3.4.4h	Thuong Tin	Ha Noi	10 February 2020	EPI_ISL_9572808
A/Muscovy duck/ Vietnam/HN6115/2020	H5N6	2.3.4.4h	Thuong Tin	Ha Noi	10 February 2020	EPI_ISL_9572794
A/Muscovy duck/ Vietnam/HN6119/2020	H5N6	2.3.4.4h	Thuong Tin	Ha Noi	10 February 2020	EPI_ISL_9572800
A/Muscovy duck/ Vietnam/HN6120/2020	H5N6	2.3.4.4h	Thuong Tin	Ha Noi	10 February 2020	EPI_ISL_9572792
A/Muscovy duck/ Vietnam/HN6606/2020	H5N6	2.3.4.4h	Gia Lam	Ha Noi	29 September 2020	EPI_ISL_9572754
A/Muscovy duck/ Vietnam/HN6607/2020	H5N6	2.3.4.4h	Gia Lam	Ha Noi	29 September 2020	EPI_ISL_9572759
A/Muscovy duck/ Vietnam/HN6608/2020	H5N6	2.3.4.4h	Gia Lam	Ha Noi	29 September 2020	EPI_ISL_9572770
A/Muscovy duck/ Vietnam/HN6609/2020	H5N6	2.3.4.4h	Gia Lam	Ha Noi	29 September 2020	EPI_ISL_9572745
A/Muscovy duck/ Vietnam/HN6610/2020	H5N6	2.3.4.4h	Gia Lam	Ha Noi	29 September 2020	EPI_ISL_9572739
A/duck/Vietnam/HN6611/2020	H5N6	2.3.4.4h	Gia Lam	Ha Noi	29 September 2020	EPI_ISL_9572741
A/Muscovy duck/ Vietnam/HN6692/2020	H5N6	2.3.4.4h	Thuong Tin	Ha Noi	4 November 2020	EPI_ISL_17809474
A/Muscovy duck/ Vietnam/HN6698/2020	H4H5N6	2.3.4.4h	Thuong Tin	Ha Noi	4 November 2020	N/A
A/Muscovy duck/ Vietnam/HN6699/2020	H4H5N6	2.3.4.4h	Thuong Tin	Ha Noi	4 November 2020	N/A
A/Muscovy duck/ Vietnam/HN6811/2020	H5H6N6	2.3.4.4h	Gia Lam	Ha Noi	30 December 2020	N/A
A/Muscovy duck/ Vietnam/HN6881/2021	H5N6	2.3.4.4h	Gia Lam	Ha Noi	29 January 2021	EPI_ISL_17809475
A/Muscovy duck/ Vietnam/HN6884/2021	H5N6	2.3.4.4h	Gia Lam	Ha Noi	29 January 2021	EPI_ISL_17809476
A/Muscovy duck/ Vietnam/HN6890/2021	H5N6	2.3.4.4h	Gia Lam	Ha Noi	29 January 2021	EPI_ISL_17809477
A/duck/Vietnam/HN6893/2021	H5N6	2.3.4.4h	Gia Lam	Ha Noi	29 January 2021	EPI_ISL_17809478
A/duck/Vietnam/HN6894/2021	H5N6	2.3.4.4h	Gia Lam	Ha Noi	29 January 2021	EPI_ISL_17809479
A/duck/Vietnam/HN6896/2021	H5N6	2.3.4.4h	Gia Lam	Ha Noi	29 January 2021	EPI_ISL_17809480
A/duck/Vietnam/HN6897/2021	H5N6	2.3.4.4h	Gia Lam	Ha Noi	29 January 2021	EPI_ISL_17809481
A/duck/Vietnam/HN6898/2021	H5N6	2.3.4.4h	Gia Lam	Ha Noi	29 January 2021	EPI_ISL_17809482
A/duck/Vietnam/QN6519/2020	H5N6	2.3.4.4h	Ha Long	Quang Ninh	21 August 2020	EPI_ISL_9572816
A/duck/Vietnam/QN6658/2020	H5N6	2.3.4.4h	Ha Long	Quang Ninh	23 October 2020	EPI_ISL_17809483
A/duck/Vietnam/QN6659/2020	H5N6	2.3.4.4h	Ha Long	Quang Ninh	23 October 2020	EPI_ISL_17809484
A/duck/Vietnam/QN6660/2020	H5N6	2.3.4.4h	Ha Long	Quang Ninh	23 October 2020	EPI_ISL_17809485
A/duck/Vietnam/QN6661/2020	H5N6	2.3.4.4h	Ha Long	Quang Ninh	23 October 2020	EPI_ISL_17809486
A/duck/Vietnam/QN6730/2020	H5N6	2.3.4.4h	Ha Long	Quang Ninh	25 November 2020	EPI_ISL_17809487
A/duck/Vietnam/QN6736/2020	H4H5N2N6	2.3.4.4h	Ha Long	Quang Ninh	25 November 2020	N/A
A/duck/Vietnam/QN6739/2020	H5N6	2.3.4.4h	Ha Long	Quang Ninh	25 November 2020	EPI_ISL_17809488
A/duck/Vietnam/QN6796/2020	H5N6	2.3.4.4h	Ha Long	Quang Ninh	25 December 2020	EPI_ISL_17809489
A/duck/Vietnam/QN6798/2020	H5N6	2.3.4.4h	Ha Long	Quang Ninh	25 December 2020	EPI_ISL_17809490
A/duck/Vietnam/QN6799/2020	H5N6	2.3.4.4h	Ha Long	Quang Ninh	25 December 2020	EPI_ISL_17809491
A/duck/Vietnam/QN6800/2020	H5N6	2.3.4.4h	Ha Long	Quang Ninh	25 December 2020	EPI_ISL_17809492
A/duck/Vietnam/QN6801/2020	H5N6	2.3.4.4h	Ha Long	Quang Ninh	25 December 2020	EPI_ISL_17809493
A/duck/Vietnam/QN6803/2020	H3H5N2N6	2.3.4.4h	Ha Long	Quang Ninh	25 December 2020	N/A
A/duck/Vietnam/QN6804/2020	H5N6	2.3.4.4h	Ha Long	Quang Ninh	25 December 2020	EPI_ISL_17809494
A/duck/Vietnam/QN6805/2020	H5N6	2.3.4.4h	Ha Long	Quang Ninh	25 December 2020	EPI_ISL_17809495
A/duck/Vietnam/QN6806/2020	H5N6	2.3.4.4h	Ha Long	Quang Ninh	25 December 2020	EPI_ISL_17809496
A/duck/Vietnam/QN6807/2020	H3H5N2N6N8	2.3.4.4h	Ha Long	Quang Ninh	25 December 2020	N/A
A/duck/Vietnam/QN6808/2020	H5N6	2.3.4.4h	Ha Long	Quang Ninh	25 December 2020	EPI_ISL_17809497
A/duck/Vietnam/QN6809/2020	H3H5N6	2.3.4.4h	Ha Long	Quang Ninh	25 December 2020	N/A
A/duck/Vietnam/HN5141/2018	H5N6	2.3.4.4g	Thuong Tin	Ha Noi	5 December 2018	EPI_ISL_17768807
A/duck/Vietnam/HN5142/2018	H5N6	2.3.4.4g	Thuong Tin	Ha Noi	5 December 2018	EPI_ISL_17768808
A/duck/Vietnam/HN5143/2018	H5N6	2.3.4.4g	Thuong Tin	Ha Noi	5 December 2018	EPI_ISL_17768809
A/duck/Vietnam/HN5144/2018	H5N6	2.3.4.4g	Thuong Tin	Ha Noi	5 December 2018	EPI_ISL_17809472
A/duck/Vietnam/HN5145/2018	H5N6	2.3.4.4g	Thuong Tin	Ha Noi	5 December 2018	EPI_ISL_17768810
A/duck/Vietnam/HN5147/2018	H5N6	2.3.4.4g	Thuong Tin	Ha Noi	5 December 2018	EPI_ISL_17768811
A/duck/Vietnam/HN5148/2018	H5N6	2.3.4.4g	Thuong Tin	Ha Noi	5 December 2018	EPI_ISL_17971917
A/duck/Vietnam/HN5149/2018	H5N6	2.3.4.4g	Thuong Tin	Ha Noi	5 December 2018	EPI_ISL_17809473

* All HA and/or NA subtypes detected by analyzing consensus sequences are listed.

## Data Availability

Sequence data presented in this study are openly available in GISAID (https://gisaid.org/). Reference numbers are provided in Table 1.

## Supplementary Materials

The following supporting information can be downloaded at: https://www.mdpi.com/article/10.3390/v15071596/s1. Supplemental Figure S1. Phylogenetic analysis of the HA genes of highly pathogenic H5N6 influenza viruses isolated in Vietnam from 2018 to 2021. Supplemental Figure S2. Phylogenetic analysis of the NA genes of highly pathogenic H5N6 influenza viruses isolated in Vietnam from 2018 to 2021. Supplemental Figure S3. Phylogenetic analysis of the PB2 genes of highly pathogenic H5N6 influenza viruses isolated in Vietnam from 2018 to 2021. Supplemental Figure S4. Phylogenetic analysis of the PB1 genes of highly pathogenic H5N6 influenza viruses isolated in Vietnam from 2018 to 2021. Supplemental Figure S5. Phylogenetic analysis of the PA genes of highly pathogenic H5N6 influenza viruses isolated in Vietnam from 2018 to 2021. Supplemental Figure S6. Phylogenetic analysis of the NS1 genes of highly pathogenic H5N6 influenza viruses isolated in Vietnam from 2018 to 2021. Supplemental Table S1: List of sequences obtained from the GISAID’s EpiFlu™ Database. Supplemental Table S2: Consensus sequences of viral proteins isolated in this study. Supplemental Table S3: Polymorphisms in the proteins of H5N6 influenza viruses isolated in Vietnam from 2018 to 2021.
