# Supplementary material for: Continued Circulation of Highly Pathogenic H5 Influenza Viruses in Vietnamese Live Bird Markets in 2018–2021
Source: Viruses. 2023 Jul 21;15(7):1596. doi: 10.3390/v15071596 (PMC10384249; doi:10.3390/v15071596)
Supplement: Supplementary file 1 [file Guan - Supplemental Figure 1 - Submitted.pdf › Guan - Supplemental Figure 1 - Submitted.pdf]

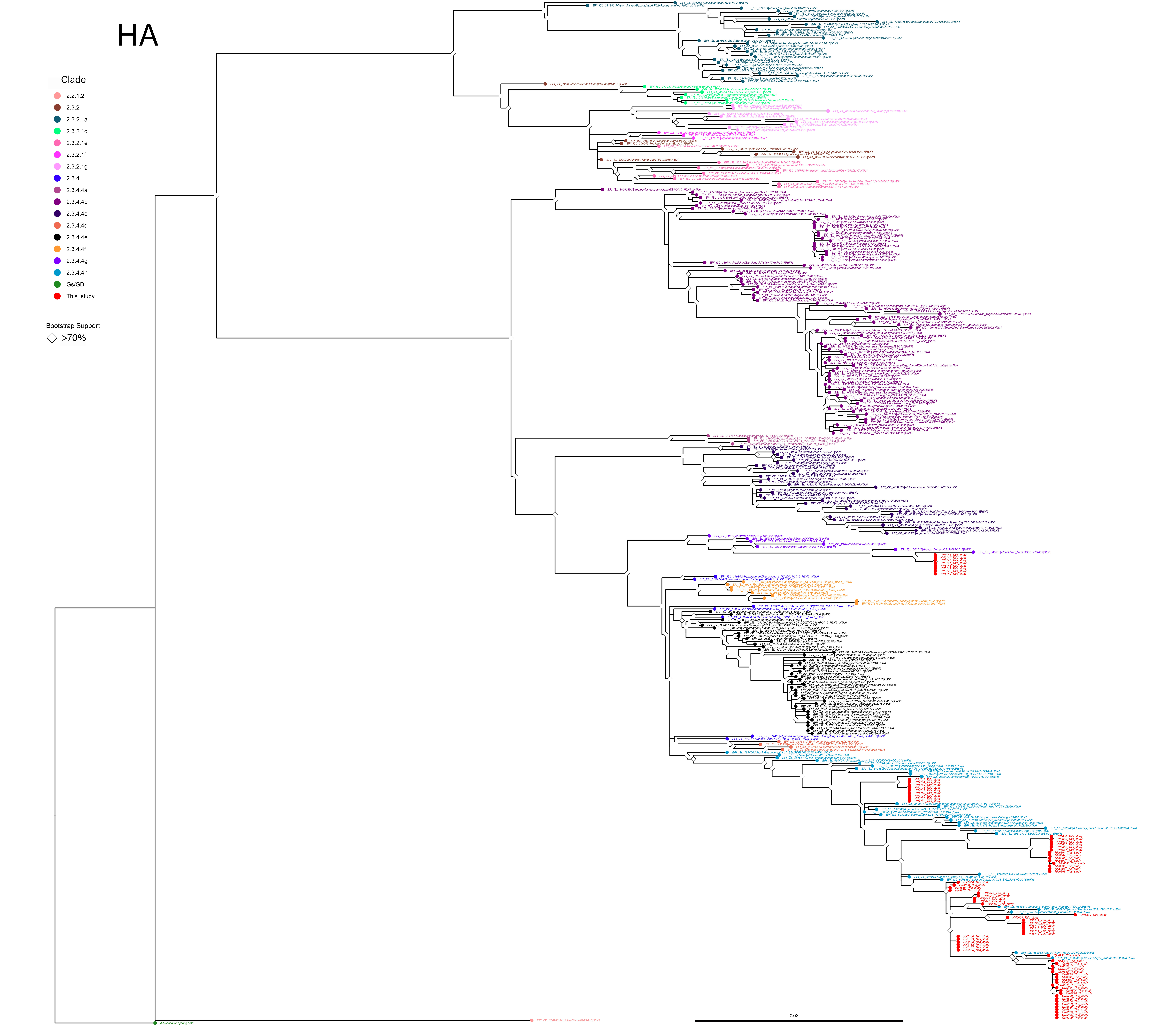
HA
Clade
2.2.1.2
2.3.2
2.3.2.1a
2.3.2.1d
2.3.2.1e
2.3.2.1f
2.3.2.1g
2.3.4
2.3.4.4a
2.3.4.4b
2.3.4.4c
2.3.4.4d
2.3.4.4e
2.3.4.4f
2.3.4.4g
2.3.4.4h
Gs/GD
This_study
Bootstrap Support
>70%
0.03
