# Supplementary material for: Continued Circulation of Highly Pathogenic H5 Influenza Viruses in Vietnamese Live Bird Markets in 2018–2021
Source: Viruses. 2023 Jul 21;15(7):1596. doi: 10.3390/v15071596 (PMC10384249; doi:10.3390/v15071596)
Supplement: Supplementary file 1 [file Guan - Supplemental Figure 2 - Submitted.pdf › Guan - Supplemental Figure 2 - Submitted.pdf]

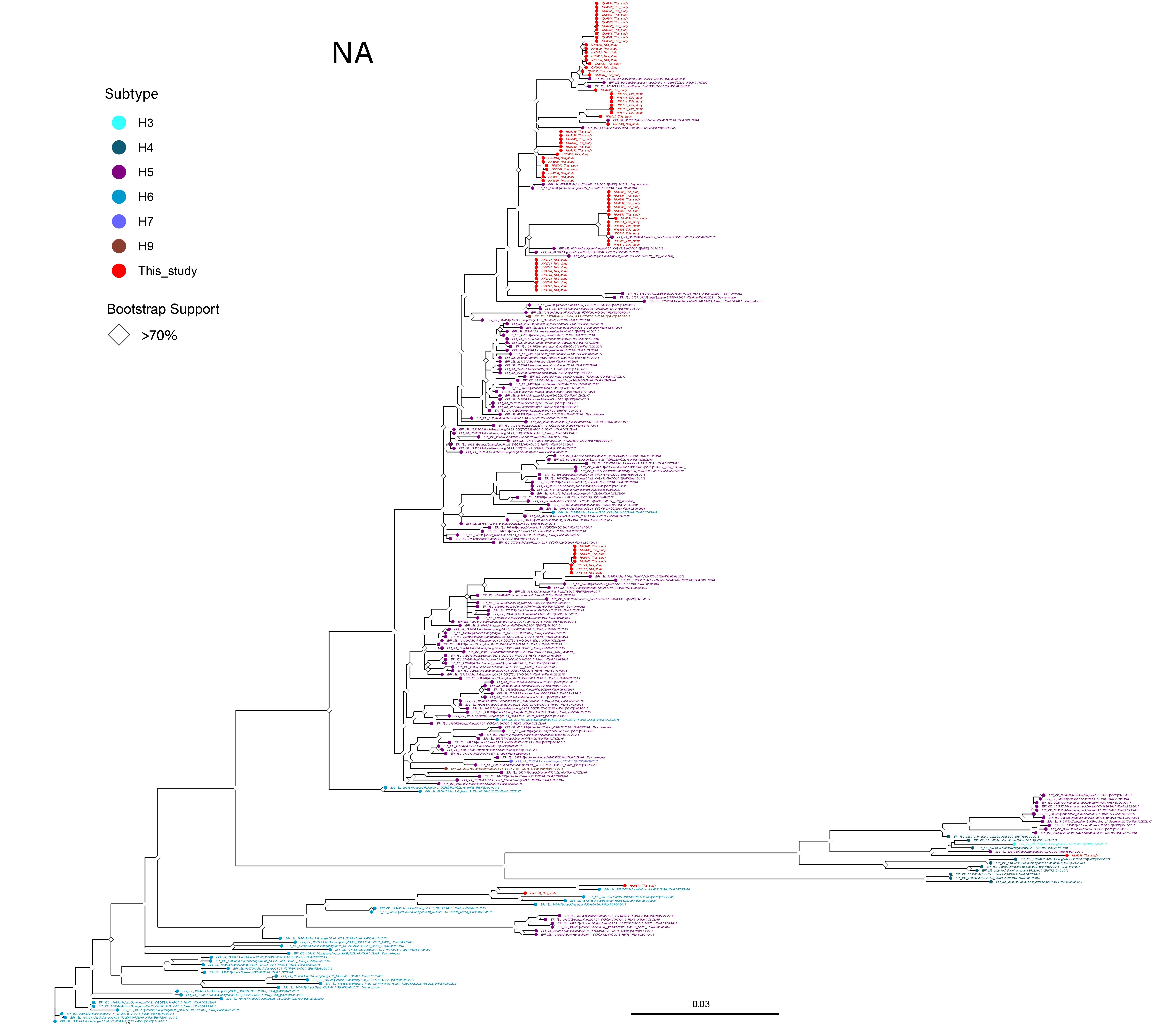
NA
Subtype
H3
H4
H5
H6
H7
H9
This_study
Bootstrap Support
>70%
0.03
