# Supplementary material for: Continued Circulation of Highly Pathogenic H5 Influenza Viruses in Vietnamese Live Bird Markets in 2018–2021
Source: Viruses. 2023 Jul 21;15(7):1596. doi: 10.3390/v15071596 (PMC10384249; doi:10.3390/v15071596)
Supplement: Supplementary file 1 [file viruses-15-01596-s001.zip › Supplementary Figures_Titles and Legends.pdf]

Supplemental Figure S1. Phylogenetic analysis of the HA genes of highly pathogenic H5N6 influenza viruses isolated in Vietnam from 2018 to 2021. The HA genes were rooted to A/goose/Guangdong/1/1996 (Gs/GD; H5N1). Viruses isolated in this study are shown in red. All other viruses are color-coded based on the HA clade. Bootstrap values greater than 70% are indicated by open diamonds. The scale bar represents the nucleotide substitution rate at each site.

Supplemental Figure S2. Phylogenetic analysis of the NA genes of highly pathogenic H5N6 influenza viruses isolated in Vietnam from 2018 to 2021. Viruses isolated in this study are shown in red. All other viruses are color-coded based on the HA subtype. Bootstrap values greater than 70% are indicated by open diamonds. The scale bar represents the nucleotide substitution rate at each site.

Supplemental Figure S3. Phylogenetic analysis of the PB2 genes of highly pathogenic H5N6 influenza viruses isolated in Vietnam from 2018 to 2021. Viruses isolated in this study are shown in red. All other viruses are color-coded based on the HA subtype. Bootstrap values greater than 70% are indicated by open diamonds. The scale bar represents the nucleotide substitution rate at each site.

Supplemental Figure S4. Phylogenetic analysis of the PB1 genes of highly pathogenic H5N6 influenza viruses isolated in Vietnam from 2018 to 2021. Viruses isolated in this study are shown in red. All other viruses are color-coded based on the HA subtype. Bootstrap values greater than 70% are indicated by open diamonds. The scale bar represents the nucleotide substitution rate at each site.

Supplemental Figure S5. Phylogenetic analysis of the PA genes of highly pathogenic H5N6 influenza viruses isolated in Vietnam from 2018 to 2021. Viruses isolated in this study are shown in red. All other viruses are color-coded based on the HA subtype. Bootstrap values greater than 70% are indicated by open diamonds. The scale bar represents the nucleotide substitution rate at each site.

Supplemental Figure S6. Phylogenetic analysis of the NS1 genes of highly pathogenic H5N6 influenza viruses isolated in Vietnam from 2018 to 2021. Viruses isolated in this study are shown in red. All other viruses are color-coded based on the HA subtype. Bootstrap values greater than 70% are indicated by open diamonds. The scale bar represents the nucleotide substitution rate at each site.
